# Transcriptional plasticity buffers genetic variation in zinc homeostasis

**DOI:** 10.1038/s41598-019-55736-0

**Published:** 2019-12-20

**Authors:** Alice Pita-Barbosa, Felipe K. Ricachenevsky, Michael Wilson, Tania Dottorini, David E. Salt

**Affiliations:** 1 Center for Coastal, Limnology and Marine Studies, Federal University of Rio Grande do Sul, Litoral Norte Campus, Imbé, RS 95625-000 Brazil; 2 Biology Department, Center of Natural and Exact Sciences, Federal University of Santa Maria, Santa Maria, Brazil; 3 Future Food Beacon of Excellence and the School of Biosciences, University of Nottingham, Nottingham, LE12 5RD UK; 4 School of Veterinary Medicine and Science, University of Nottingham, Nottingham, LE12 5RD UK

**Keywords:** Plant physiology, Natural variation in plants, Plant molecular biology

## Abstract

In roots of *Arabidopsis thaliana*, Zn can be either loaded into the xylem for translocation to the shoot or stored in vacuoles. Vacuolar storage is achieved through the action of the Zn/Cd transporter *HMA3* (*Heavy Metal Atpase 3*). The Col-0 accession has an *HMA3* loss-of-function allele resulting in high shoot Cd, when compared to accession CSHL-5 which has a functional allele and low shoot Cd. Interestingly, both Col-0 and CSHL-5 have similar shoot Zn concentrations. We hypothesize that plants sense changes in cytosolic Zn that are due to variation in HMA3 function, and respond by altering expression of genes related to Zn uptake, transport and compartmentalisation, in order to maintain Zn homeostasis. The expression level of genes known to be involved in Zn homeostasis were quantified in both wild-type Col-0 and Col-0::*HMA3*^*CSHL-5*^ plants transformed with the functional CSHL-5 allele of *HMA3*. We observed significant positive correlations between expression of *HMA3* and of genes known to be involved in Zn homeostasis, including *ZIP3, ZIP4, MTP1*, and *bZIP19*. The results support our hypothesis that alteration in the level of function of *HMA3* is counterbalanced by the fine regulation of the Zn homeostasis gene network in roots of *A. thaliana*.

## Introduction

Zinc (Zn) is an essential element to plants. While Zn deficiency can cause stunted growth and decreased productivity, excessive Zn concentration in plant tissues can lead to toxicity. Therefore, plants evolved mechanisms to tightly control Zn uptake, tissue distribution and subcellular compartmentalization to maintain proper Zn homeostasis^1,2^. The main gene families implicated in Zn homeostasis are ZIP (Zinc-regulated/Iron-regulated Protein), which perform primary Zn uptake into cells^3,4^; Metal Tolerance Protein (MTP), involved in Zn detoxification into vacuoles^5^; ZIFL (Zinc-Induced Facilitator-Like), which are able to transport nicotianamine and phytosiderophores that bind Zn^6^; and HMA (Heavy Metal-Associated), with members that act as Zn efflux transporters from the cytoplasm into the vacuole or to the apoplast^7–9^. Yellow-Stripe Like proteins, which transport metal-phytosiderophore/nicotianamine complexes, are also thought to have a role in Zn homeostasis^10^. Moreover, the transcription factors bZIP19 and bZIP23 from *A. thaliana* were shown to be important in the Zn deficiency response^11^.

Plants may also accumulate elements with no known biological function, such as cadmium (Cd). High Cd concentration in leaves and seeds can make plants a source of Cd exposure for humans, and since Cd is a class I carcinogen, avoiding Cd accumulation in the edible tissue of plants is a key factor for food safety^12,13^. Thus, studies dissecting Cd transport in plants have described a number of key genes, especially in genetically tractable species such as *A. thaliana* and rice^14,15^. Importantly, it was shown that Cd transporters localized in root vacuolar membranes compartmentalize Cd in the vacuole, leading to lowered Cd availability in the root symplast, and lower xylem loading. Thus, increased accumulation of Cd into root vacuoles decreased the Cd concentration in leaves and seeds^16,17^. Examples of transporters capable of compartmentalising Cd into root vacuoles includes in *A. thaliana* AtABCC1 and AtABCC2, a duplicated gene pair that is able to transport Cd-phytochelatin complexes into root vacuoles^18,19^; the CAX-type proton antiporters, AtCAX2 and AtCAX4 from *A. thaliana*^20,21^; and AtHMA3^22^.

The vacuolar transporter AtHMA3 and its orthologs in other species were shown to control Cd concentration in above-ground tissues. In *A. thaliana*, loss-of-function of *AtHMA3* resulted in increased Cd sensitivity, whereas ectopic over-expression improved tolerance, establishing its role in Cd detoxification through Cd accumulation in root vacuoles^22^. AtHMA3 was shown to control natural variation in leaf Cd in *A. thaliana* with functional *AtHMA3* alleles conferring low leaf Cd concentrations, whereas accessions with non-functional alleles showed increased leaf Cd concentrations^7^. Interestingly, Col-0, the most commonly used *A. thaliana* accession, has a non-functional truncated *AtHMA3* sequence. Transformation of Col-0 with the genomic fragment containing a functional allele of *AtHMA3* resulted in decreased leaf Cd concentration, demonstrating that root vacuole compartmentalization affects root-to-shoot translocation^7^. Similarly, in rice *OsHMA3* natural variation was shown to control Cd accumulation in shoots and grains, with functional alleles restricting Cd root-to-shoot translocation by enhancing storage of Cd in root vacuoles, and with loss-of-function alleles showing the opposite phenotype^9,23,24^. Further, in *Brassica rapa BjHMA3* has been established to control natural variation in leaf accumulation of Cd, similar to both *A. thaliana* and rice, with polymorphisms in the coding region of *BjHMA3* driving variation in function^25^. Allelic variation of *BrHMA3*, shown to affect leaf Cd accumulation in *B. rapa*, was observed to have no effect on leaf Zn concentration. *OsHMA3* ectopic over-expression resulted in increased Cd tolerance and lower Cd concentration in leaves and grains, but increased Cd concentration in roots^23,26^. Thus, HMA3 function is likely conserved in *A. thaliana*, *B. rapa* and rice, and is a major determinant controlling Cd movement from roots to above-ground tissues.

Limited evidence suggests that *AtHMA3* and *OsHMA3* also have a role in Zn homeostasis. Although *AtHMA3* does not complement a yeast mutant lacking the Zn vacuolar transporter *zrc1*^27^, *A. thaliana* loss-of-function mutant *athma3* shows slightly increased Zn sensitivity, whereas plants ectopically over-expressing *AtHMA3* show increased Zn tolerance^22^. However, genetic transformation of *A. thaliana* Col-0, which contains a non-functional allele of *AtHMA3*, with a functional allele of *AtHMA3* from the *A. thaliana* CSHL-5 genotype only changed Zn leaf concentration very slightly, suggesting that any functional impact of AtHMA3 was potentially buffered by other mechanisms to maintain shoot Zn concentration in the Col-0 accession^7^. Similarly, in rice ectopic over-expression of *OsHMA3* did not significantly change shoots Zn concentrations. Maintenance of Zn homeostasis in shoots was attributed to increased expression of ZIP transporters in roots of the over-expressing line. Since Zn is an essential element, regulatory mechanisms may be activated to increase uptake in lines that have an enhanced function of HMA3 that is driving increased Zn compartmentalisation into root vacuoles^26^. However, these results in rice were observed in lines that were ectopically over-expressing *OsHMA3* from the maize *ubiquitin1* promoter, which also drives expression in shoots. This may not be a relevant physiological context, given that shoot expression of normally root expressed Zn transporters is known to produce unexpected results on Zn homeostasis^28^. In a recently published study, rice genotypes with functional *OsHMA3* were compared with genotypes containing natural loss-of-function alleles, and the authors observed that plants with functional OsHMA3 were more tolerant to high Zn concentration and able to accumulate more Zn in roots, while maintaining similar Zn concentrations in shoots. This was due to up-regulation of Zn uptake genes in roots of genotypes with higher expression of *HMA3*^29^. However, the data is based on four genotypes, and it is possible that other genetic factors are involved in the correlations observed. Thus, the question of whether HMA3 is a component of the Zn homeostasis network in plants, and further how this network is reorganized to accommodate varying levels of HMA3 function, remain to be answered.

In this work, we used previously reported transgenic Col-0 lines that have been transformed with a functional *AtHMA3* allele, isolated from the CSHL-5 *A. thaliana* accession (collected from Cold Spring Harbor Lab, Long Island, NY), and expressed from its native promoter. We used these lines to address the question of how plants with an increased root vacuolar Zn sink, driven by enhanced activity of HMA3, regulate Zn homeostasis. We performed experiments using a hydroponic system to identify changes in expression of genes in roots known to be involved in Zn homeostasis. We found that variation in *AtHMA3* expression in our lines is correlated with expression of key genes in the Zn regulatory gene network, providing a direct causal relationship between HMA3 activity and the status of the Zn transcriptional regulatory network. Our work highlights how *A. thaliana* plants can transcriptionally buffer Zn homeostasis when Zn compartmentalization in root vacuoles is altered through the modification of AtHMA3 activity.

## Results

### Zn leaf concentration is not associated with genetic variation in AtHMA3

**Figure 1:**
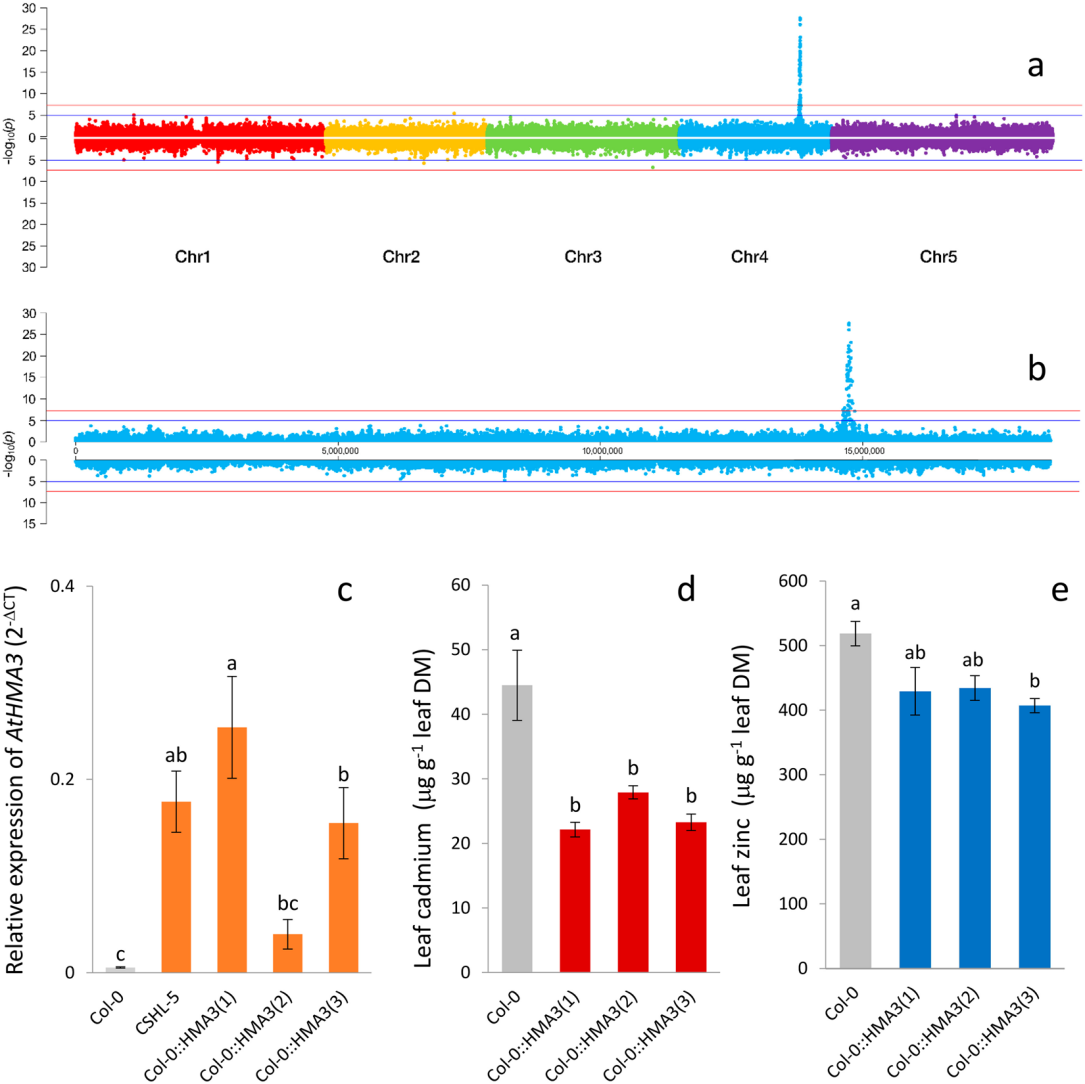
Genome-wide association mapping of leaf cadmium (top) and zinc (bottom) of 206,088 SNPs across 340 *A. thaliana* accessions using a mixed model analysis. Horizontal blue line indicates suggestive significance (-log_10_(1e-5)), horizontal red line indicates genome-wide significance (−log_10_(1e-7.5)) **(a)**. Chromosome 4 detail **(b)**. Relative expression of *AtHMA3* in roots of *A. thaliana* Col-0, CSHL-5 and Col-0::HMA3^CSHL−5^ lines. *PEX4* and *EF1-α4* were used as internal normalization standard across all samples and *HMA3* expression levels were calculated by 2^−ΔCT^ method^52^ **(c)**. Concentration of cadmium **(d)** and zinc **(e)** in leaves. Bars correspond to the means of biological replicates and the error bars correspond to the standard error. Letters above bars indicate statistically significant differences using a one-way ANOVA with Tukey’s test using a 95% confidence interval.

Previously, we conducted genome wide association (GWA) studies using leaf Cd concentration and genotype data derived from the 256 K SNP-tilling array Atsnptile 1, containing 248,584 SNPs^30^, for 337 *A. thaliana* accessions^7^. We found a single peak in chromosome 4 with multiple SNPs associated with leaf Cd concentration (Fig. 1A). The causative gene was identified as *AtHMA3*, which was shown to be a major driver of variation in leaf Cd concentration in *A. thaliana* species-wide diversity^7^. Since evidence suggests that AtHMA3 transports Zn as well as Cd into vacuoles, we hypothesized that genetic variation in *AtHMA3* could also be associated with leaf Zn concentration. We conducted GWA mapping using the genotypic and Zn leaf data from the same experiment as described in Chao *et al*.^7^. However, we found no SNPs significantly associated with variation in Zn in the region of *AtHMA3* (Fig. 1B). Thus, functional/hypofunctional *AtHMA3* alleles do not seem to control variation in leaf Zn concentration in the same way they control leaf Cd concentration.

One possible explanation for this intriguing finding is that plasticity in the Zn transcriptional network buffers the impact of AtHMA3 on Zn. In contrast, because plants do not require Cd, it is not sensed and therefore does not trigger transcriptional buffering. In order to test this hypothesis we used previously generated transgenic *A. thaliana* lines of the Col-0 accession, which has a naturally truncated, non-function allele of *AtHMA3*, and which we had transformed with the genomic fragment containing the functional *AtHMA3* allele from the CSHL-5 *A. thaliana* accession (hereafter Col-0::*HMA3*^*CSHL-5*^), including its promoter, coding sequence and 3′ UTR^7^. We expect these lines to show *AtHMA3* expression in tissues and at levels similar to the native *AtHMA3* gene from CSHL-5. Variation in expression level should be derived from positional effects of transgene insertion and number of copies inserted.

We evaluated the expression of *AtHMA3* in Col-0, CSHL-5 and the three transgenic homozygous lines of Col-0::*HMA3*^*CSHL-5*^ (lines #1, #2, and #3) (Fig. 1C). Across the transgenic lines, #3 showed the highest relative expression level: 2.5 and 4.2 times higher than #1 and #2 respectively. This range of variation in *AtHMA3* expression in the Col-0::*HMA3*^*CSHL-5*^ transgenic lines allowed use to effectively test the impact of *AtHMA3* function on the expression of genes involved in Zn homeostasis. Expression of the *HMA3*^*CSHL-5*^ allele in our three Col-0 transgenic lines was also found to be of a similar magnitude to its native expression level in CSHL-5 (Fig. 1C).

The concentration of Cd in leaves of these transgenic lines was approx. 45% lower than in Col-0, with all three lines showing similar decreases in Cd concentration (Fig. 1D). Zn concentrations, on the other hand, were only slightly reduced in the Col-0::*HMA3*^*CSHL-5*^ genotype compared to Col-0, and only line #3 showing a statistically significant reduction (Fig. 1E). Interestingly, line #3 showed the highest *AtHMA3* expression level among the transgenic lines (Fig. 1C). We found no change in concentrations of other elements in the Col-0::*HMA3*^*CSHL-5*^ genotype (Table S1). These observations establish the impact of AtHMA3 activity on both Cd and Zn concentration in leaves, reproducing in hydroponically cultivated plants that observed by Chao *et al*.^7^ in plants cultivated in soil.

### Modulation of AtHMA3 activity leads to changes in expression of Zn homeostasis genes in roots

We next tested whether modulation of AtHMA3 function in the Col-0 accession induces transcriptional changes in known Zn-related genes in root tissues. We evaluated expression levels of *AtZIP1*, *AtZIP2*, *AtZIP3*, *AtZIP4*, *AtZIP5*, *AtbZIP19*, *AtHMA4*, *AtNAS1*, *AtYSL2*, *AtPCR2*, *AtMTP1*, *AtMTP3* and *AtZIF1* in all three Col-0::*HMA3*^*CSHL-5*^ lines. Regression analyses including only the complemented lines (i.e., we excluded non-transgenic Col-0 plants expression data) were used to identify any possible relationship between the expression of *AtHMA3* and transcription of each selected gene.

**Figure 2:**
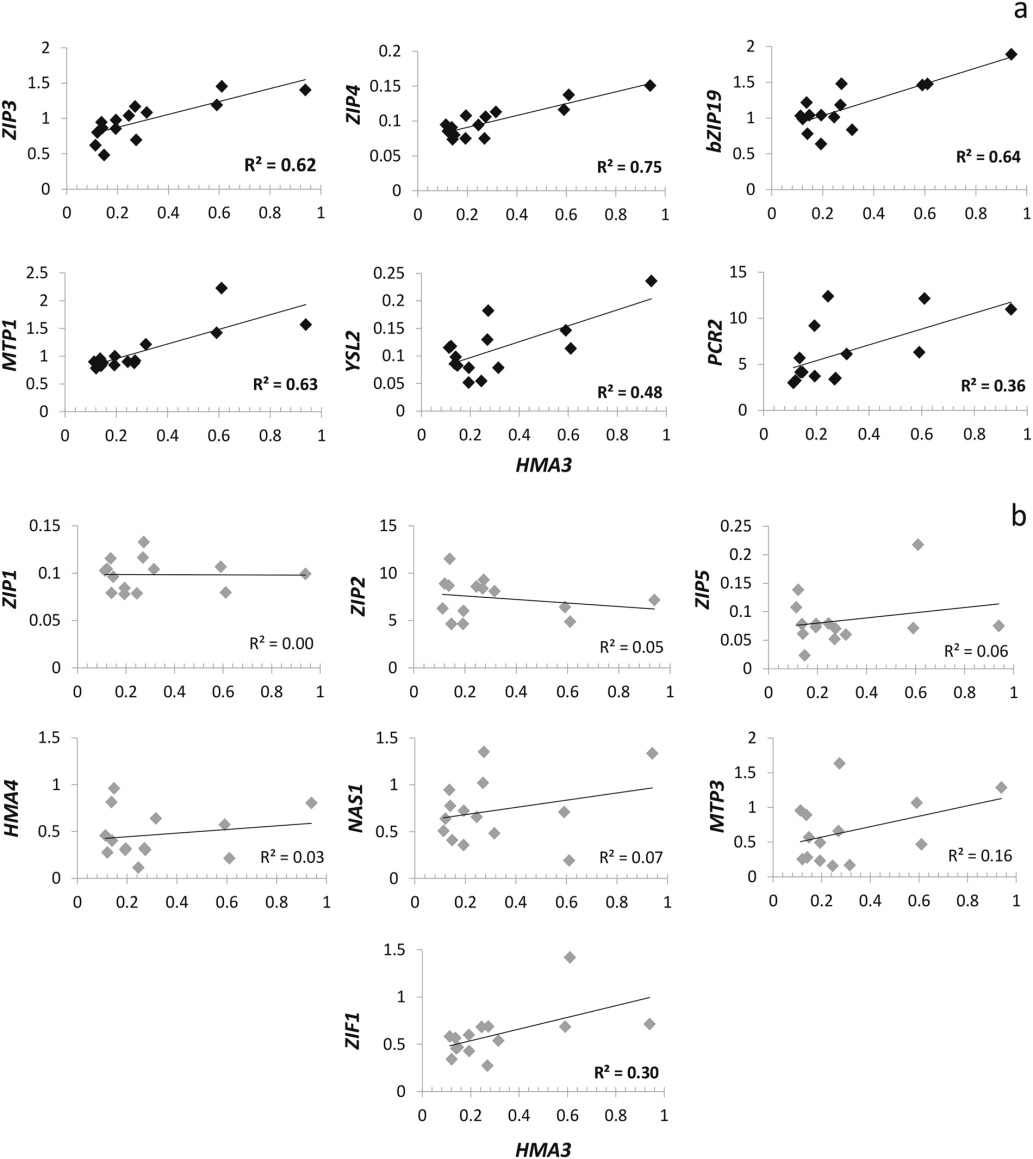
Correlation between root relative expression of *AtHMA3* and Zn-related genes in transgenic lines. *PEX4* and *EF1-α4* were used as internal normalization standard across all samples and the expression levels were calculated by 2^−ΔCT^ method^52^. The points in Fig. 2 refer to individual plants from the three complementation lines used in this work. Significant correlations (*P ≤ 0.05; **P ≤ 0.01; ***P ≤ 0.001) are shown as black diamonds (**a**) and non-significant correlations are shown as grey diamonds (**b**).

We found several genes involved in Zn homeostasis where the expression of the gene in roots was significantly correlated with the increasing expression of *AtHMA3*. These genes were the Zn plasma membrane transporters *AtZIP3* and *AtZIP4*; the Zn effluxer *AtPCR2*; the vacuolar Zn transporter *AtMTP1*; and the putative Fe and Zn-phytosiderophore transporter *AtYSL2* (Fig. 2A). Expression of the transcription factor *AtbZIP19*, which was shown to bind the *AtZIP4* promoter, and is important for uptake under Zn deficiency, also showed a positive correlation with *AtHMA3* expression (Fig. 2A). *AtZIP1*, *AtZIP2*, *AtZIP5*, *AtHMA4*, *AtNAS1*, *AtMTP3* and *AtZIF1* were also tested, but showed no significant correlation with *AtHMA3* expression (Fig. 2B).

**Figure 3:**
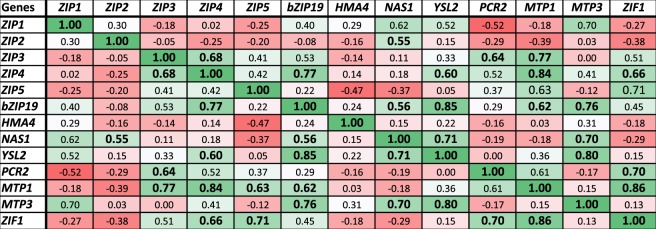
Pearson’s correlation analysis among Zn-related genes used as target in the qPCR analysis. Significant correlations (*P* ≤ 0.05) are represented in bold.

After identifying Zn homeostasis genes that show altered expression in response to *AtHMA3* expression, we performed a Pearson’s correlation analysis to find other possible interactions across the genes studied. We found a significant strong correlation (r ≥ 0.7) between expression of *AtZIP3* x *AtMTP1*, *AtZIP4* x *AtbZIP19*, *AtZIP4* x *AtMTP1*, *AtZIP5* x *AtZIP1*, *AtbZIP19* x *AtYSL2*, *AtbZIP19* x *AtMTP3*, *AtMTP1* x *AtZIF1*, *AtMTP3* x *AtNAS1*, *AtMTP3* x *AtYSL2*, *AtNAS1* x *AtYSL2*, *AtPCR2* x *AtZIF1*. This result and other significant correlations (0.56 ≤ r ≤ 0.69, classified here as moderate) are shown in Fig. 3.

**Figure 4:**
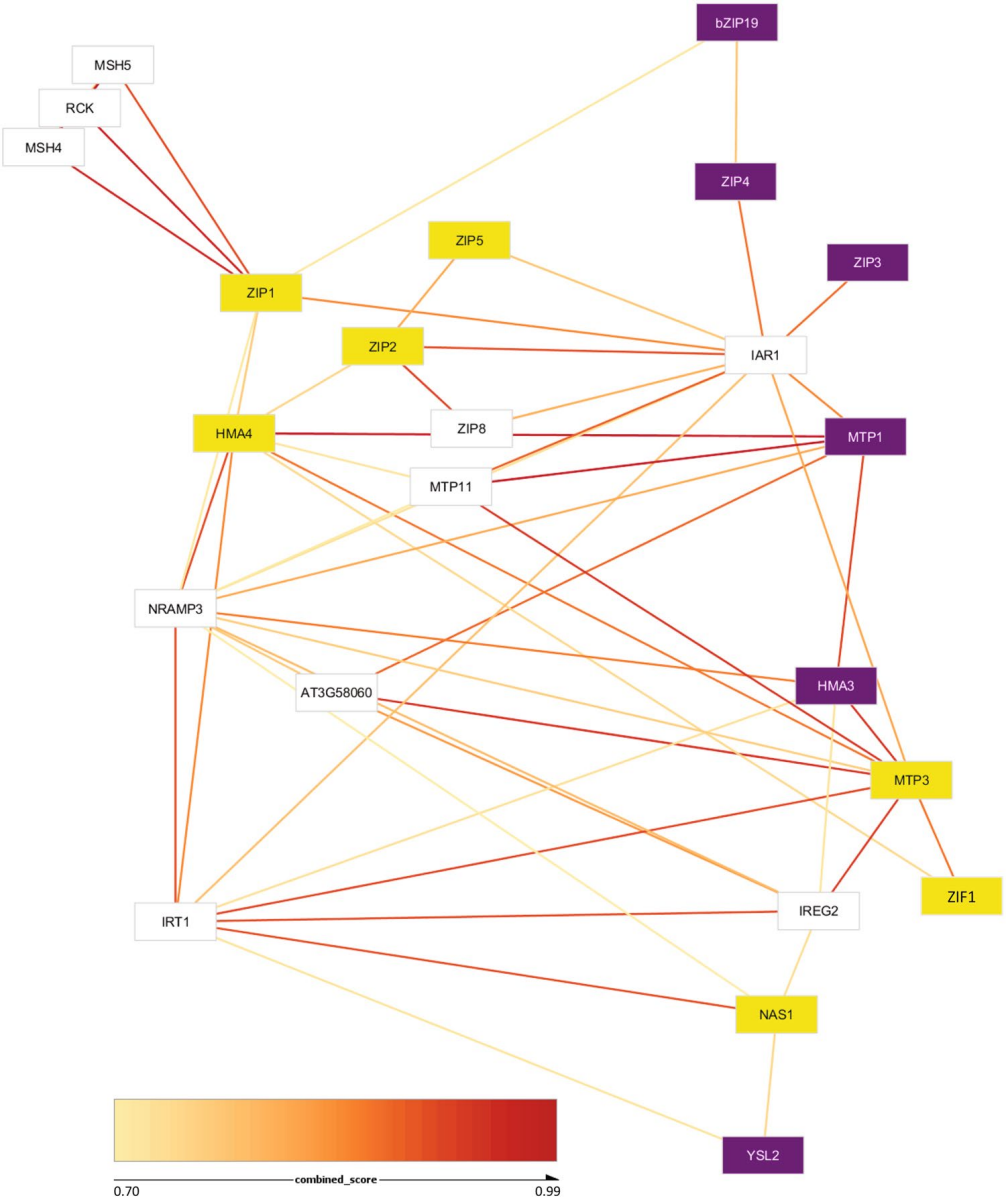
Predicted Protein-Protein Association (PPFA) network performed using the STRING platform. The purple rectangles are proteins whose gene expression is significantly correlated with that of *AtHMA3* and the yellow rectangles show proteins whose gene expression is not significantly correlated with that of *AtHMA3*, based on the real-time qPCR expression analysis performed in the present work. The white boxes represent proteins with predicted associations in STRING, but not assayed in this work.

To support the correlation analyses based on gene expression presented above, we also investigated the Protein-Protein Functional Associations (PPFA) network of AtHMA3. This network was generated using various public data sources from the STRING^31^ platform, relating to the 14 target genes we monitored in this study to see if the proteins we found to be correlated had any predicted functional associations with AtHMA3, either directly or indirectly. The PPFA network corroborated most of the results of the gene expression analyses: AtHMA3 is predicted to be functionally associated directly with AtMTP1, which in turn is associated with AtZIP3 and AtZIP4 through AtIAR1 (Fig. 4). AtZIP4, in turn is functionally associated with AtbZIP19. However, our experimental analysis differs from the PPFA network in that *AtHMA3* expression is not correlated with *AtZIP1/2/5*, *AtHMA4*, *AtNAS1*, and *AtMTP3* expression (Fig. 2b). Taken together, our correlation and PPFA network data support the conclusion that functional changes in AtHMA3 alter the expression of genes that are part of the transcriptionally co-regulated Zn homeostasis network.

## Discussion

In *A. thaliana*, almost 2400 proteins have Zn-related functions^32^, including enzymes associated with antioxidant systems (Cu/Zn superoxide dismutase)^33^ and photosynthesis (carbonic anhydrase)^34^. Thus, cellular Zn concentration needs to be finely tuned. There is evidence that the vacuolar localised HMA3 transports Zn^9,22,29^, and that natural functional allelic variation exists for *HMA3* in *A. thaliana*, rice and *B. rapa*^7,24,25,35^. Intriguingly, this variation in HMA3 functions appears to have very limited impact on Zn accumulation, and is primarily manifest as variation in Cd accumulation^7,24–26,29,35^. One plausible explanation for this is that changes in the transcriptional networks regulating Zn homeostasis buffer variation in *HMA3* function. Here, we show that *A. thaliana* plants do transcriptionally buffer changes in Zn compartmentalization in root vacuoles as a direct consequence of modification of *AtHMA3* function, in order to maintain a constant Zn concentration in leaves. Further, our data provides a clear answer to the previously posed question of ‘why plants expressing the functional form of the Cd/Zn transporter *AtHMA3* (such as CSHL-5 genotype) show lower Cd concentration in the leaves compared to genotypes with non-functional *AtHMA3* (such as Col-0), while Zn concentration is not significantly changed’^7^?

A recent study using rice natural accessions with functional and non-functional *OsHMA3* alleles suggested that Zn homeostasis related genes have their steady-state level changed when OsHMA3 activity varies in order to maintain Zn concentrations in shoots^29^. Accessions with a functional *OsHMA3* allele showed higher expression levels of *OsZIP4*, *OsZIP5*, *OsZIP8*, and *OsZIP10* in roots compared to accessions with a non-functional *OsHMA3* allele^29^. These results from Cai *et al*.^29^ agree with data from *OsHMA3* over-expressing lines, which have decreased Cd concentration in shoots, but similar Zn concentrations compared to WT, which was attributed to up-regulation of *OsZIP4*, *OsZIP5*, *OsZIP8*, *OsZIP9* and *OsZIP10*^26^. However, experiments with over-expressing lines can be misleading, since expression at high levels in all cells is not physiologically relevant. Experiments with natural accessions, although informative, should also be interpreted carefully, since natural accessions can vary at many genetic loci, making it difficult to draw conclusions about the contribution of any given locus from a small selection of accessions such as explored by Cai *et al*.^29^.

In our experiment with *A. thaliana*, we used the Col-0 accession which has a naturally occurring non-functional allele of *AtHMA3*, and transformed this accession with a functional allele from the CSHL-5 *A. thaliana* accession under control of its native promoter, to generate multiple independent Col-0::*HMA3*^*CSHL-5*^ lines, which were compared to wild type Col-0 plants as a control. In this experimental set up, the only genetic variation between the lines is the presence/absence of the functional *AtHMA3* allele and its expression levels due to insertional effects and copy number variation. Using this variation in expression of the functional CSHL-5 *AtHMA3* allele in the null *hma3* Col-0 background, we were able to directly test the impact of AtHMA3 function on the Zn homeostasis transcriptional network.

We found that expression of functional *AtHMA3* alleles in a background with no functional *AtHMA3* promotes changes in expression of multiple genes involved in Zn homeostasis in roots. ZIP genes (*AtZIP3* and *AtZIP4*; Fig. 2A) were upregulated. *AtZIP3* is predominantly expressed in roots of Zn-deficient plants, whereas *AtZIP4* is induced by Zn deficiency in both roots and shoots^36^. Induction of ZIP transporters was also observed in rice ectopically overexpressing a functional *OsHMA3* allele, compared to genotypes with a loss-of-function allele^26^. Both can complement yeast mutants that are defective in Zn uptake^3,11^, indicating their role in maintaining Zn homeostasis, although their precise physiological role is unclear. Interestingly, two highly similar transcription factors essential for Zn deficiency responses in *A. thaliana*, AtbZIP19 and AtbZIP23, can bind to Zinc Deficiency Response Elements (ZDRE) in the promoter of both *AtZIP4* and *AtZIP3*, and promote up regulation of expression^11,37^. Lilay *et al*.^38^ proposed that Zn modulated the activity of AtbZIP19 at the protein level, in the nucleus, where cellular Zn sufficiency represses its activity. Importantly, we found *AtbZIP19* expression to be positively correlated with *AtHMA3* expression (Fig. 2A), indicating that the higher the AtHMA3 function, the more the plants induce Zn deficiency-related genes, supporting our conclusion that increased compartmentalization of Zn into vacuoles induces expression of key Zn-related genes in order to maintain cytosolic Zn homeostasis.

The expression of functional *AtHMA3* also showed a high correlation to *AtMTP1* (Metal Tolerance Protein 1), related to Zn-excess. The AtMTP1 transporter is located at the vacuolar membrane, and is known to be involved in the detoxification of excess Zn through its compartmentalisation into vacuoles^39,40^. We also found correlation between *AtHMA3* and *AtPCR2* in our transgenic lines. AtPCR2 is a Zn exporter with a dual function, effluxing Zn into the rhizosphere and xylem under Zn excess^41^. Thus, increased expression of *AtMTP1* and *AtPCR2* upon higher AtHMA3 activity suggests that roots are sensing local Zn excess when HMA3 function is high. It also indicates that increased AtHMA3 function is not enough to sequester Zn in the vacuole when Zn uptake is higher due to increased expression of AtZIP3/AtZIP4.

**Figure 5:**
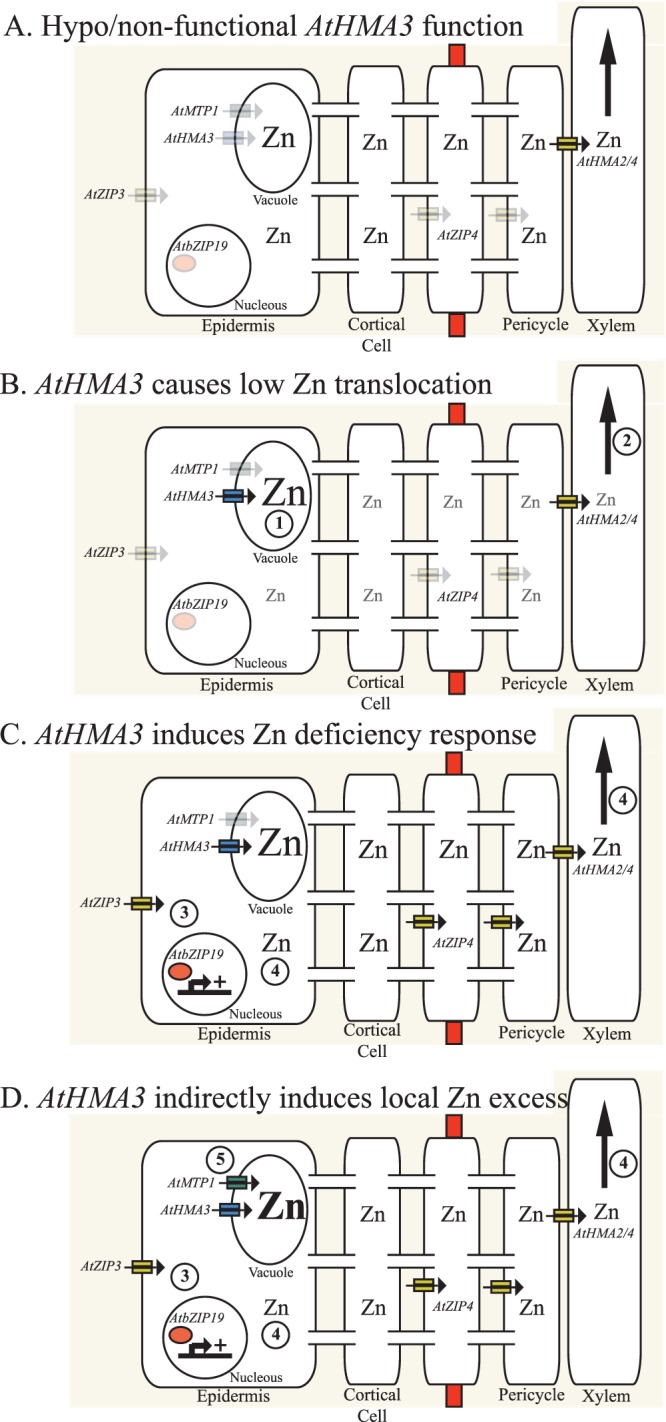
Working model of how AtHMA3 function changes the Zn homeostasis transcriptional network. (**A**) Zn homeostasis in roots of plants with hypo-functional/non-functional AtHMA3. Zn in the symplast is maintained low, and Zn is also stored in the root vacuoles. (**B**) Zn homeostasis changes in roots of plants with higher AtHMA3 function. Higher AtHMA3 function results in (1) higher vacuolar Zn storage, and a consequent lowering of Zn symplastic concentration, which result in (2) decreased Zn root-to-shoot translocation and decreased cytosolic Zn concentration. (**C**) As a result of decreased Zn root-to-shoot translocation and presumable lower Zn concentration in shoots and/or roots, (3) Zn uptake transporters are up regulated, which (4) re-establish Zn symplastic concentration, Zn xylem and therefore maintain Zn shoot concentration. (**D**) As a result of sustained up regulation of Zn uptake transporters, roots might undergo local, transient Zn excess in the cytoplasm, which explain (5) up regulation of Zn vacuolar detoxification transporters. The order of event may be different than presented. Changes in ‘Zn’ font size represent higher/lower Zn concentrations. Changes in the transparency of the text represent variation in the expression of transporters (less transparent more expression).

We propose that functional copies of *AtHMA3* result in increased Zn transport into root vacuoles. Cytosolic Zn availability decreases, leading to less Zn translocation from roots to shoots. Zn uptake transporters (*AtZIP3* and *AtZIP4*) are up regulated, which could be a response to a shoot-derived signal^28^ or to lower local Zn status^2^. Increased Zn uptake leads to higher local Zn concentration in the root symplast, which causes increased expression of Zn excess responsive genes (Fig. 5). This transcriptional buffering of the Zn homeostasis network in response to variation in AtHMA3 function maintains Zn concentration in shoots. The expression simultaneously of both Zn deficiency and Zn excess genes in response to increasing vacuolar Zn compartmentalization (driven by AtHMA3) is reminiscing of the ‘zinc – shock’ hypothesis proposed in yeast where the vacuolar Zn transport gene *ZRC1* is upregulated under Zn-limiting conditions^42^. Our model presented in Fig. 5 shows how *A. thaliana* plants would acclimate to increased Zn vacuolar sequestration upon higher HMA3 function. It is important to acknowledge that the model is based on the correlation analyses, and needs further functional validation.

The model proposed in Fig. 5 is supported by the STRING-based PPFA Network that allowed us the identification of functional associations between *AtHMA3* and all the genes we show experimentally to be co-regulated with *AtHMA3*, either through direct or indirect associations. This network analysis also shows that the experimentally determined *AtHMA3* driven co-regulation network (that includes *bZIP19*, *ZIP4*, *ZIP3*, *MTP1* and *YSL2*) is a subnetwork of a more complete Zn homeostasis network that also links into iron (Fe) and manganese (Mn) homeostasis, through such genes as *IRT1* (high affinity iron uptake transporter^43^), and *MTP8* (AT3G58060; vacuolar Mn transporter^44,45^).

Zn homeostasis is finely controlled through complex transcriptional buffering regulated by Zn sensing, and this buffering is able to compensate for alterations in the strength of the vascular Zn sink in roots. However, this is not the case for Cd, where leaf Cd concentration is strongly affected by alteration in the strength of the root vascular Cd sink. We conclude that this difference between Zn and Cd is due to the fact that plants are not able to sense Cd in the same way as Zn. They are therefore unable to modulate transcriptional networks to buffer leaf Cd in the same way they can for Zn. This leads to the intriguing idea that Cd can act as a ‘tracer’ to reveal hidden genetic variation in the Zn homeostatic network. A clear example of this is the use of Cd accumulation to identify functional variation in the vacuolar Zn transporter HMA3 in *A. thaliana*, *B. rapa*, and rice^7,9,23–25^.

## Methods

### Plant materials and growth conditions

Seeds from *A. thaliana* Col-0 and three lines expressing the functional allele of *HMA3* from CSHL-5 (collected from Cold Spring Harbor Lab, Long Island, NY) in the Col-0 background (Col-0::HMA3^CSHL-5^) were cultivated for 4 weeks in nutrient solution containing 10% Hoagland solution in which Fe was replaced by Fe-HBED [N,N9-di(2-hydroxybenzyl)ethylenediamine-N,N9-diacetic acid monohydrochloride hydrate; Strem Chemicals, Inc.]. We also added a non-toxic Cd concentration of 0.05 μM [as Cd(NO_3_)]. The plants were kept in a climate-controlled room for growth with a photoperiod of 10 h light (100 mmol m^−2^ s^−1^)/14 h dark, humidity of 60% and temperature ranging from 19 to 22 °C. After 4-weeks growth, roots were harvested and used for RNA extraction and shoots were harvested for elemental analysis, performed as described by Chao *et al*.^7^. We performed two independent experiments using a randomized block design, and similar results were obtained. Here we show the results of one experiment, which had five biological replicates for Col-0 and transgenic lines Col-0::HMA3(1) and Col-0::HMA3 (2), and four biological replicates for line Col-0::HMA3 (3).

### Genome-wide association mapping

GWA mapping was performed using the GWA-Portal^46^. Elemental concentrations of Cd and Zn were determined by ICP-MS as described here and in Chao *et al*.^7^, for 340 accessions from the core set in that work. The 340 accessions were genotyped using the custom-designed SNP-tilling array Atsnptile 1^30^, resulting in 206,088 SNPs between both phenotype and genotyped accession sets. The GWA analysis was done using an accelerated mixed model implemented in the program AMM, described previously^47^.

### Selection of target genes

The following genes which expression has been previously shown to be regulated by Zn deficiency in *A. thaliana* were chosen for this study: *bZIP19*^11^, *ZIF1*^6,40^, *ZIP1*^3,11,48,49^, *ZIP3*^11,36^, *ZIP4*^11,36,49^, and *ZIP5*^11^. We have also evaluated the expression of *ZIP2*^36^, *HMA4*^8^, *MTP1*^28,39,50^*, MTP3*^51^, *NAS1*^6^, *PCR2*^41^, and *YSL2*^49^ genes, due to their known functions in Zn homeostasis.

### Quantitative real-time PCR

Total RNA was extracted from roots using TRIzol Plus RNA Purification kit (Invitrogen Life Technologies). After DNA removal with DNAse I (Invitrogen), two micrograms of total RNA was used to synthesize first strand cDNA with Transcriptor First Strand cDNA Synthesis Kit (Roche). Quantitative real-time PCR was performed using SensiFAST^TM^ SYBR® Hi-ROX Kit (Bioline), using the first strand cDNA as a template, on a Real-Time PCR System (ABI StepOnePlus, Applied Biosystems, USA). Primers for qRT-PCR were designed to cover an exon-exon junction using the Primer-BLAST platform (NCBI). Primer sequences are shown in Table S2. For the analysis, *PEX4* (AT5G25760) and *EF1-α4* (AT5G60390) were both used as internal normalization standards across all samples. Expression levels were calculated using the 2^−ΔCT^ method^52^. For all samples analysed primer efficiencies were corrected using the Miner algorithm^53^.

### Protein-protein functional association network

The PPFA for the 14 *A. thaliana* genes (*AtHMA3*, *AtZIP1*, *AtZIP2*, *AtZIP3*, *AtZIP4*, *AtZIP5*, *AtbZIP19*, *AtHMA4*, *AtNAS1*, *AtYSL2*, *AtPCR2*, *AtMTP1*, *AtMTP3* and *AtZIF1*) were obtained from STRING v11.0^31^. The network is built on the association score calculated by STRING, which are calculated based on the following sources of evidence: text-mining, neighbourhood, experiments, gene fusion, databases, co-occurrence and co-expression. We highlight that associations do not necessarily mean that proteins are physically binding each other, but jointly contributing to a shared function. We generated our network using a minimum association score of 0.7 (high confidence) and a maximum number of 10 associations for the first shell, and export to cytoscape v3.7. AtPCR2 was excluded from the final analysis because it had no associations in our network.

### Statistical analysis

The experiment was conducted using a randomized block design, with five Col-0 and 14 transgenic Col-0::HMA3^CSHL-5^ plants (biological replicates) derived from 3 independent Col-0::HMA3^CSHL-5^ lines. We used five replicate plants for lines Col-0::HMA3(1) and Col-0::HMA3(2), and four plants for line Col-0::HMA3(3). The investigation of differences in *AtHMA3* expression and leaf Zn and Cd between the genotypes was carried out using the ANOVA F-test (*p* ≤ 0.05) test. The correlation between expression of *AtHMA3* and the target genes was evaluated using linear regression. We performed normality tests for all variables and residues of regression, and the p-value was corrected using the Benjamini-Hochberg test (P ≤ 0.023). Pearson’s correlation coefficient was used to estimate correlations across all the target genes tested in this study.

## Supplementary information

Supplementary Material
