# Supplementary material for: Transcriptional plasticity buffers genetic variation in zinc homeostasis
Source: Sci Rep. 2019 Dec 20;9:19482. doi: 10.1038/s41598-019-55736-0 (PMC6925235; doi:10.1038/s41598-019-55736-0)
Supplement: Supplementary file 1 — Supplementary Material [file 41598_2019_55736_MOESM1_ESM.pdf]

Transcriptional plasticity buffers genetic variation in zinc homeostasis

Alice Pita-Barbosa[1,3], Felipe K. Ricachenevsky[2], Michael Wilson[3], Tania Dottorini[4] and David E. Salt[3]*

Table S1. Ionomics profile of *Arabidopsis thaliana* Col-0 and Col-0::HMA3[CSHL-5] lines (one-way ANOVA, $P \leq 0.05$)

Element	ANOVA	Sum of Squares	df	Mean Square	F	Sig.
As	BG	0.00951539	3	0.0031718	0.6564	0.5914
	WG	0.072481	15	0.00483207		
	Total	0.0819964	18	0.5882		
B	BG	0.0198491	3	0.00661637	0.1925	0.8999
	WG	0.515583	15	0.0343722		
	Total	0.535432	18	0.8995		
Ca	BG	0.00543574	3	0.00181191	0.3318	0.8025
	WG	0.0819034	15	0.00546023		
	Total	0.0873392	18	0.8055		
Co	BG	0.0137991	3	0.00459969	1.439	0.2735
	WG	0.044744	14	0.003196		
	Total	0.0585431	17	0.2717		
Cu	BG	0.0292428	3	0.00974761	2.046	0.1537
	WG	0.0667121	14	0.00476515		
	Total	0.0959549	17	0.1533		
Fe	BG	0.0711234	3	0.0237078	1.557	0.2553
	WG	0.167527	11	0.0152297		
	Total	0.23865	14	0.1827		
K	BG	0.0179175	3	0.00597249	1.559	0.2407
	WG	0.0574652	15	0.00383101		
	Total	0.0753826	18	0.2381		
Li	BG	0.00419364	3	0.00139788	0.06185	0.9791
	WG	0.339018	15	0.0226012		
	Total	0.343212	18	0.9805		
Mg	BG	0.00146501	3	0.000488336	0.1428	0.9327
	WG	0.051281	15	0.00341873		
	Total	0.052746	18	0.9312		
Mn	BG	0.00911051	3	0.00303684	1.148	0.3621
	WG	0.0396936	15	0.00264624		
	Total	0.0488041	18	0.3624		
Mo	BG	0.033977	3	0.0113257	2.924	0.07071
	WG	0.0542227	14	0.00387305		
	Total	0.0881997	17	0.07173		
Na	BG	0.279709	3	0.0932364	3.057	0.07359
	WG	0.335455	11	0.0304959		
	Total	0.615164	14	0.07848		
Ni	BG	0.0111789	3	0.00372631	0.4315	0.7335
	WG	0.129535	15	0.00863566		
	Total	0.140714	18	0.7766		
P	BG	0.0107989	3	0.00359964	0.9971	0.4211
	WG	0.0541539	15	0.00361026		
	Total	0.0649528	18	0.4202		
Rb	BG	0.0170098	3	0.00566994	1.018	0.4122
	WG	0.0835124	15	0.0055675		
	Total	0.100522	18	0.4115		
S	BG	0.00877547	3	0.00292516	1.223	0.3358
	WG	0.0358841	15	0.00239228		
	Total	0.0446596	18	0.332		
Se	BG	0.00517408	3	0.00172469	0.3758	0.7718

	WG	0.0688477	15	0.00458985		
	Total	0.0740218	18	0.7717		
Sr	BG	0.0119539	3	0.00398462	1.529	0.2477
	WG	0.0390778	15	0.00260518		
	Total	0.0510316	18	0.2426		

BG: between groups; WG: within groups; Sig.: significance.

Table S2. Primers used for gene expression analysis by qRT-PCR

Gene	*Locus*	*Primer pairs (5′ → 3′)**
bZIP19 (Transcription Factor bZIP19)	AT4G35040	(+)CGTGCTTCCATGTCCACACC<br>(-)CCCGGTTTCCCAAAGGTCTC
EF1-a4 (Elongation Factor 1-alpha 4)**	AT5G60390	(+)CACATTTTCGTAGCCGCAAGACTCC<br>(-)GATGACACGCTTGTCAATACCACC
HMA3 (Heavy Metal ATPase3)	AT4G30120	(+)TGCTGCTCATAAGGCAAGCA<br>(-)CTAGTCCAGTATCCGCGATCACT
HMA4 (Heavy Metal ATPase4)	AT2G19110	(+)GCTCAAGAACAGCTAGGGAATG<br>(-)CATTGCGGTTGGTCCCTCTT
MTP1 (Metal Tolerance Protein 1)	AT2G46800	(+)CTCCCTGCGGATGTAATCCATT<br>(-)GAGATTGACGCCACAAAGCTC
MTP3 (Metal Tolerance Protein A2)	AT3G58810	(+)TTTGCCTTCCATCCCGAA<br>(-)CTGATGTTGCAGCCTTTGCA
NAS1 (Nicotianamine Synthase 1)	AT5G04950	(+)CATGATCTTCCACACAACGGAC<br>(-)CGACGTCATATTGGTCAAGGC
PCR2 (Plant Cadmium resistance 2)	AT1G14870	(+)ACAAAACCTTACATTGCTTTCCATT<br>(-)TCCTTCAGCATGAGGCTTGG
PEX4 (Peroxin 4)**	AT5G25760	(+)CTGCGACTCAGGGAATCTTCTAA<br>(-)TTGTGCCATTGAATTGAACCC
YSL2 (Yellow Stripe Like 2)	AT5G24380	(+)CTTATCCTAGTGGAACGGCAAC<br>(-)TCTGTTTCTTGGCTGTCTTATCTCC
ZIF1 (Zinc Induced Facilitator 1)	AT5G13740	(+)TTGCTATCTCAGGTCTCGGC<br>(-)GTATCATCAGTGCCCCAGCA
ZIP1 (Zinc Transporter 1)	AT3G12750	(+)AGGAAAGAGAGCAAAGAGGCAA<br>(-)TCCCAGCAGATGCAGCATT
ZIP3 (Zinc Transporter 3)	AT2G32270	(+)AAAGCGTCTAAGAGGGTCAG<br>(-)TATTCCCAACTCCAATACCTGTG
ZIP4 (Zinc Transporter 4)	AT1G10970	(+)TTCTCAGATATTGGAGCTCGG<br>(-)ATGGAGACTGTGATACCCCC
ZIP5 (Zinc Transporter 5)	AT1G05300	(+)TCCTCGCAACGGGGTTTATG<br>(-)CCTGTGAACGGAAACTCCCA

* (+) and (-) indicate forward and reverse primers, respectively.

** Genes used as endogenous control.
